# Subclinical myocardial dysfunction in treatment naive papillary thyroid carcinoma patients

**DOI:** 10.1038/s41598-026-41816-5

**Published:** 2026-03-06

**Authors:** Safak Akin, Gokhan Giray Akgul, Mehmet Ali Gulcelik, Murat Celik, Uygar Cagdas Yuksel, Cevdet Erdol, Nese Ersoz Gulcelik

**Affiliations:** 1 Gülhane Faculty of Medicine, Department of Endocrinology and Metabolism, University of Health Sciences, Ankara, Turkey; 2 Department of Molecular Endocrinology, University of Health Sciences, Gülhane Institute of Health Sciences, Ankara, Turkey; 3 Gülhane Faculty of Medicine, Department of Surgical Oncology, University of Health Sciences, Ankara, Turkey; 4 Gülhane Faculty of Medicine, Department of Cardiology, University of Health Sciences, Ankara, Turkey; 5 Department of Cardiology, University of Health Sciences, Ankara, Turkey

**Keywords:** Papillary thyroid carcinoma, Left ventricular diastolic dysfunction, Global longitudinal strain, Integrin αvβ3, Thyroid feedback quantile-based index, Biomarkers, Cardiology, Diseases, Medical research

## Abstract

Papillary thyroid carcinoma (PTC) may contribute to cardiovascular (CV) morbidity through pro-inflammatory signaling and endothelial dysfunction, yet subclinical myocardial effects remain poorly characterized. While cardiovascular morbidity in papillary thyroid carcinoma (PTC) patients has largely been attributed to treatment-related factors such as TSH suppression, the potential contribution of the disease itself to early, subclinical cardiovascular vulnerability has not been well defined. To address this knowledge gap, we examined treatment-naïve PTC patients for evidence of subclinical cardiovascular dysfunction. This case-control study included 36 untreated PTC patients and 20 benign nodular goiter controls. Comprehensive cardiac assessment utilized transthoracic echocardiography, tissue Doppler imaging, and two-dimensional speckle-tracking echocardiography. Serum integrin αvβ3 and tumor necrosis factor-alpha (TNF-α) levels were quantified via enzyme-linked immunosorbent assay. Central thyroid hormone sensitivity was assessed using the Thyroid Feedback Quantile-based Index (TFQI). Compared with controls, patients with PTC exhibited evidence of subclinical myocardial functional alterations. Circulating integrin αvβ3 and TNF-α levels were significantly higher in patients with PTC. Integrin αvβ3 levels were associated with impaired GLS and diastolic dysfunction, whereas TNF-α showed no direct association with echocardiographic parameters. In addition, TFQI_FT4_ levels were increased in PTC group and demonstrated a positive correlation with circulating integrin αvβ3 levels, as well as an association with impaired GLS. Treatment-naive, euthyroid patients with PTC exhibit early subclinical myocardial dysfunction despite preserved ejection fraction. Impairment in GLS, derived from echocardiographic assessment, is associated with circulating integrin αvβ3 and altered central thyroid hormone sensitivity, reflecting early CV alterations that warrant confirmation in longitudinal studies.

## Introduction

Well-differentiated thyroid carcinoma (DTC), comprising papillary and follicular thyroid cancer, demonstrates an excellent prognosis with appropriate treatment and a 5-year mortality rate of less than 1%^1^. However, emerging evidence suggests an increased risk of cardiovascular (CV) complications in DTC patients, including higher incidence of atrial fibrillation (AF), heart failure (HF), and coronary artery disease (CAD)^2–4^. Notably, DTC patients exhibit a 3.3-fold higher risk of CV mortality and a 4.4-fold higher risk of all-cause mortality compared to controls, independent of traditional CV risk factors^5^. A meta-analysis of 193 320 DTC patients confirmed significantly increased risks of AF, CAD, and overall mortality^6^, though some studies report conflicting results^7^. These discrepancies highlight the complex relationship between DTC and CV risk, influenced by confounding factors including thyroid function status, medication effects, and prior treatments.

The contribution of malignant processes to cardiovascular disease (CVD) development in DTC patients remains poorly understood. With excellent survival rates, understanding CV morbidity has become essential for optimizing long-term management strategies. Despite advances in cardiac imaging technologies and the growing recognition of diastolic dysfunction as an early predictor of adverse CV outcomes^8^, a comprehensive cardiac assessment has not yet been conducted in newly diagnosed patients with papillary thyroid carcinoma (PTC). To elucidate these potential cardiac effects, we employed two-dimensional speckle-tracking echocardiography (2D-STE), a sensitive modality capable of detecting early subclinical myocardial dysfunctions. Specifically, global longitudinal strain (GLS) was used to assess subtle impairments in left ventricular systolic function that may precede conventional echocardiographic abnormalities^9,10^. Importantly, diastolic dysfunction often precedes systolic impairment and serves as a reliable early marker of myocardial involvement across various clinical settings. Tissue Doppler imaging complements this evaluation by providing detailed insights into left ventricular relaxation and filling pressures^11^. Thus, the integration of advanced echocardiographic techniques is essential for uncovering the early effects of PTC on CV function, offering valuable implications for both pathophysiological understanding and clinical risk stratification.

Various molecular markers have been investigated in thyroid cancer^12,13^, among which integrin αvβ3, a plasma membrane receptor expressed on proliferating endothelial, cancer, and cardiac fibroblast cells, has gained attention as a potential molecular link between thyroid cancer and CVD^14–18^. This receptor functions as a non-genomic thyroid hormone receptor, mediating pro-angiogenic signaling and promoting tumor-associated angiogenesis. In vitro studies demonstrate that papillary and follicular thyroid cancer cell proliferation depends on integrin αvβ3-mediated pathways^19^. In parallel with these molecular insights, recent advances in thyroid homeostasis assessment have introduced thyroid hormone sensitivity indices, which provide more accurate thyroid function evaluation than traditional TSH and free thyroxine (FT4) measurements^20^. Among these, the Thyroid Feedback Quantile-based Index (TFQI) has been linked to both thyroid cancer^21^ and CVD^22^. However, the relationship between altered central thyroid hormone sensitivity and integrin αvβ3, as well as the manner in which these factors may coexist with increased CV vulnerability, has not yet been examined in PTC.

Therefore, the present study aimed to investigate early myocardial functional abnormalities in newly diagnosed euthyroid PTC patients using echocardiographic assessment, and to examine their associations with circulating integrin αvβ3 levels and indices of central thyroid hormone sensitivity.

## Methods

### Participants

This cross-sectional case-control study was conducted at the Department of Endocrinology and Metabolism, Gülhane Faculty of Medicine, between July 31, 2021, and July 31, 2022. From a total of 225 patients assessed during this period, 56 participants were selected based on predefined inclusion and exclusion criteria. Inclusion criteria were age ≥ 18 years and normal thyroid function tests, while exclusion criteria included history of thyroid dysfunction (hypo- or hyperthyroidism), previous thyroid surgery, established CVDs, diabetes mellitus, severe hepatic or renal failure, connective tissue disorders, previous malignancy, pregnancy or lactation, or use of medications affecting thyroid or cardiac function (including corticosteroids, levothyroxine, or anti-thyroid drugs). Hypertension and dyslipidemia were not used as exclusion criteria at the time of patient enrollment. None of the participants were receiving lipid-lowering therapy, while two patients in the PTC group and one patient in the benign nodular goiter (BNG) group were on single-agent antihypertensive therapy; blood pressure was well controlled at the time of evaluation.

The study comprised 36 patients with newly diagnosed PTC without distant metastasis and 20 patients with BNG. All participants were consecutively recruited from the outpatient clinic. PTC diagnosis was confirmed by final histopathological examination, while the BNG group was matched to the PTC group for age, gender, and body mass index (BMI). Comprehensive medical histories were obtained from all participants, followed by thorough physical examinations to identify atypical symptoms and exclude structural heart disease. All participants were evaluated in accordance with the American Thyroid Association (ATA) guidelines^23^.

### Data collection

Demographic, clinical, and laboratory data were collected. Body weight, height, waist circumstance, and blood pressure were measured according to standard protocols. Body mass index was calculated as body weight (kg) divided by the square of the body height (m^2^). Body composition parameters of the participants were evaluated after the first voiding in the morning and with light clothes using a segmental body composition analyzer (BC-418, Tanita Corp., Tokyo, Japan). Serum levels of TSH, FT4, and FT3 were measured with the automated immunochemiluminescent assay (ICMA) kits (Roche GmbH, Mannheim, Germany). The Coefficients of variation for these thyroid profile assays were all below 10%. Euthyroid was defined as TSH (0.38–5.33.38.33 mIU/L), FT4 (0.58–1.38.58.38 ng/dL), and FT3 (2.0–4.4.0.4 pg/mL) within the reference ranges.

### Carotid artery intima-media thickness

Carotid artery intima-media thickness (CIMT) was assessed using B-mode ultrasound (MyLabSeven: Esaote) with a linear-array transducer operating at a frequency of at least 7 MHz by an observer blinded to the participant’s clinical data. Participants were examined in the supine position with the head slightly extended and rotated away from the observer. Right and left carotid artery segments were scanned longitudinally (common, proximal internal carotid arteries and the carotid bulb) through B-mode grayscale imaging. CIMT was measured from images of the distal 1 cm of the far wall of common carotid artery, between the intimal luminal and the medial-adventitial interfaces of the carotid artery wall. Three measurements at three sites of the common carotid artery were averaged to assess mean CIMT. The mean value of the right and left mean CIMT measurements was used in the analysis.

### Echocardiographic examination

Transthoracic 2D-ECHO recordings of all participants in our study were obtained using a Philips Epiq 7 (Philips Medical Systems, Bothell, WA) system equipped with a 3.5 MHz transducer. Images were acquired after expiration, ensuring that each recording included at least three consecutive cardiac cycles in the left lateral decubitus position, to minimize artifacts caused by respiratory or cycle-to-cycle variability and to ensure reliable data acquisition. Two experienced operators blinded to the participants’ clinical details conducted the 2D-ECHO evaluations to ensure unbiased data interpretation. Echocardiographic examinations were performed according to the criteria of the American Society of Echocardiography (ASE) guidelines^24^. The following parameters were assessed on the M-mode recordings: left ventricular end-diastolic diameters (LVEDd), left ventricular posterior wall thickness end diastole (LVPWd), interventricular septum thickness end diastole (IVSd), and left atrial diameter (LAD). Left ventricular (LV) systolic function was evaluated using the ejection fraction (EF) calculated according to the Teichholz formula^24^. LV mass (LVM) was calculated by Devereux’s formula^25^ and indexed to the body surface area (BSA). LV hypertrophy was defined as LVM index (LVMI) > 120 g/m^2^ for men and > 116 g/m^2^ for women^26^. Doppler studies provided indexes of ventricular filling derived from the mitral inflow velocity curves at both the early diastolic phase (E wave) and the peak late diastolic flow (A wave), as well as the E/A ratio. In addition, the isovolumic relaxation time (IVRT) and deceleration time (DT) were measured. LV diastolic function was evaluated by E wave, A wave, E/A ratio and also DT and IVRT.

### Tissue Doppler imaging

Tissue Doppler imaging was performed using transducer frequencies of 3.5–4.0.5.0 MHz. The spectral pulsed Doppler signal filters were adjusted until a Nyquist limit of 15–20 cm/sec was achieved and the minimal optimal gain was used. Myocardial velocities named as peak systolic (S_m_), early diastolic (E_m_), and late diastolic (A_m_) were obtained at the lateral mitral and lateral tricuspid annulus.

### 2D speckle tracking echocardiography analysis

Left ventricular GLS was measured using 2D-STE to detect early myocardial deformation even when traditional echocardiographic parameters, such as left ventricular ejection fraction, remain normal. For this purpose, apical 2-, 3-, and 4-chamber views were obtained and analyzed offline with a commercial software (QLAB 13, TOMTEC/Philips, Andover, MA, USA), following established professional guidelines. The mean GLS was measured by averaging the peak GLS values of apical four-, three-, and two- chamber images. A 17-segment polar plot (Bulls’ eye), which uses a color-coded display of peak systolic strain values, was employed to provide a detailed evaluation of segmental myocardial function. This comprehensive approach provided both global and regional insights into cardiac performance, facilitating a thorough understanding of myocardial mechanics and structural adaptations.

### Integrin αvβ3 and TNF-α analysis

Fasting blood samples were collected from each participant between 8:00 and 10:00 a.m., then immediately centrifuged at 3000 RPM for 10 minutes and stored at −80°C until analysis. Serum levels of integrin αvβ3 and TNF-α were measured using commercial enzymatic immunoassay kits, following the manufacturer’s instructions (BT Lab, Korain Biotech). Serum integrin αvβ3 levels were reported in ng/mL, with a sensitivity of 0.094 ng/mL and serum TNF-α levels were reported in ng/L, with a sensitivity of 1.52 ng/L. The inter- and intra-assay coefficients of variation for all three kits were below 10%.

### Index of central thyroid hormone sensitivity

The index of central thyroid hormone sensitivity included the Thyroid Feedback Quantile-based Index (TFQI). TFQI_FT4_ was calculated using the algorithm TFQI= cdfFT4- (1-cdfTSH) developed by Laclaustra et al^20^. The index range is ± 1. Negative values indicate that the hypothalamus-pituitary-thyroid (HPT) axis is more sensitive to changes in THs, whereas positive values indicated lower sensitivity to thyroid hormones. TFQI_FT4_ reflects central thyroid hormone feedback regulation rather than circulating thyroid hormone concentrations and should therefore be interpreted as a state marker of central thyroid hormone sensitivity.

### Statistical analysis

Statistical analysis was performed using SPSS for Windows version 26.0. The Kolmogorov-Smirnov and Shapiro-Wilk tests were used to evaluate the distribution pattern of numerical data. Continuous variables with normal distributions were expressed as mean $\pm$ standard deviation (SD) while categorical variables were presented as frequencies (%). Student’s t-test was used for the mean comparison of parametric variables and Mann Whitney test was used if the distribution was nonparametric. Chi-square tests were used to compare categorical variables. Pearson’s or Spearman test was used for parametric and non-parametric correlations, respectively. To determine the independent variables likely to affect the GLS, a multivariate linear regression analysis was performed. Moreover, logistic regression analysis model was used and confounders were adjusted to explore the correlation between integrin $\rm{\alpha }$ v $\upbeta$ 3 levels and the indecence of LV diastolic dysfunction. A significance level of P ≤ 0.05 was considered to indicate a statistically significant difference. The sample size of the study was determined using the G^*^Power 3.1.9.4 program. A priori minimum required sample size was calculated based on an alpha of 0.05, a power of 80%, and 20 individuals for each group. The a priori sample size calculation was performed for between-group comparisons of primary echocardiographic parameters and was not intended to support complex multivariable modeling. The post-hoc power analysis revealed that the study achieved a statistical power of 98% for integrin αvβ3 (Cohen’s d=1.14) and 94% for GLS (Cohen’s d=0.99) at an alpha level of 0.05, confirming the of the primary comparisons despite the group imbalance.

### Ethical considerations

The study was performed in line with the principles of the Declaration of Helsinki. Approval was granted by the Ethics Committee of Gülhane Training and Research Hospital (Date 24.03.2021/No 2021/6). Written informed consent was obtained from all participants before enrollment.

## Results

### Patient characteristics

The clinical and laboratory characteristics of all study participants are summarized in Table 1. By design, euthyroid BNG patients and those with PTC were well matched with respect to age, gender, BMI, body surface area, and waist-to-hip ratio. There were no statistically significant differences in systolic and diastolic blood pressure, heart rate, and CIMT between both groups (Table 1). In addition, biochemical characteristics did not differ between the two groups (Table 1). Among the PTC patients, 19.4% had tumors smaller than 1 cm, multifocality was present in 47.2%, minimal extrathyroidal extension was observed in 11.1%, lymph node metastasis was detected in 38.9%, and the majority of patients were classified as stage I disease (88.9%).

**Table 1:** Characteristics of the study population.

**Variable**	**Papillary thyroid cancer (n=36)**	**Benign nodular goiter (n=20) **	**P value**
Age (years)	44.83 $\pm$ 14.10	49.45 $\pm$ 7.14	0.178
Gender (F/M)	27/9	16/4	0.470
BMI (kg/m^2^)	28.17 $\pm$ 6.24	28.08 $\pm$ 4.04	0.949
BSA (m^2^)	1.88 $\pm$ 0.28	1.79 $\pm$ 0.19	0.196
WHR	0.87 $\pm$ 0.09	0.85 $\pm$ 0.06	0.404
SBP (mmHg)	115.94 $\pm$ 9.85	122.14 $\pm$ 10.36	0.067
DBP (mmHg)	72.39 $\pm$ 8.39	75.92 $\pm$ 8.53	0.199
CIMT (mm)	0.38 $\pm$ 0.04	0.38 $\pm$ 0.05	0.865
HR (bpm)	76.94 $\pm$ 10.2	74.92 $\pm$ 10.16	0.567
FBG (mg/dL	96.27 $\pm$ 37.49	92.07 $\pm$ 11.38	0.548
Creatinine (mg/dL)	0.86 $\pm$ 0.15	0.81 $\pm$ 0.09	0.177
Total cholesterol (mg/dL)	193.19 $\pm$ 28.57	200.36 $\pm$ 28.22	0.573
LDL-C (mg/dL)	122.33 $\pm$ 25.32	118.43 $\pm$ 20.18	0.430
HDL-C (mg/dL)	52.97 $\pm$ 13.98	56.14 $\pm$ 11.36	0.405
Triglyceride (mg/dL)	120.30 $\pm$ 62.79	113.14 $\pm$ 44.58	0.654
TSH (µIU/mL)	2.44 $\pm$ 1.40	2.15 $\pm$ 1.11	0.395
FT4 (pmol/L)	10.96 $\pm$ 2.39	11.20 $\pm$ 1.47	0.632
FT3 (pmol/L)	5.62 $\pm$ 0.63	5.18 $\pm$ 0.96	0.085
TFQI_FT4_	−0.33 $\pm$ 0.09	−0.39 $\pm$ 0.09	0.036
Integrin $\rm{\alpha }$ v $\upbeta$ 3 (ng/mL)	24.27 $\pm$ 8.01	15.47 $\pm$ 7.18	<0.001
TNF-$\rm{\alpha }$ (ng/L)*	271.68 (29.5–752.5.5.5)	194.5 (6.11–704.07.11.07)	0.021

Abbreviations: BMI, Body Mass Index; BSA, Body Surface Area; WHR, Waist to Hip Ratio; SBP, Systolic Blood Pressure; DBP, Diastolic Blood Pressure; CIMT, Carotid-Intima Media Thickness; HR, Heart Rate; FBG, Fasting Blood Glucose; LDL-C, Low-Density Lipoprotein Cholesterol; HDL-C, High-Density Lipoprotein Cholesterol; TSH, Thyroid-stimulating Hormone; FT4, Free Thyroxine; FT3, Free Triiodothyronine; TFQI_FT4_, Thyroid Feedback Quantile-Based Index calculated by FT4; TNF-$\alpha$, Tumor Necrosis Factor- $\alpha$. * median (minimum-maximum), Mann-Whitney U test.

### Cardiovascular assessment

The echocardiographic results are reported in Table 2. The mean EF was similar in both groups. No differences were observed in the two-dimensional measurements in the PTC group compared with BNG group. Regarding LV diastolic function, patients with PTC showed impaired diastolic function as demonstrated by decreased E/A ratio and the prolonged DT. With reference to the study by Nagueh et al.^27^, grade 1 diastolic dysfunction was present in 20 (55.6%) in the PTC group and 5 (25%) in the BNG group (P = 0.028) (Table 2). Within the study cohort, no cases of grade II or grade III diastolic dysfunction were observed.

**Table 2:** Comparison of echocardiographic parameters of the study population.

Variable	Papillary thyroid cancer (n=36)	Benign nodular goiter (n=20)	P value
2D echocardiography			
LVM (g)	135.01±33.79	128.98±32.63	0.516
LVMI (g/m^2^)	71.79±14.45	63.80±17.57	0.092
IVSd (mm)	9.27±1.19	9.67±1.28	0.258
LVPWd (mm)	8.73±1.16	8.96±1.10	0.470
LVEDd (mm)	45.23±3.17	42.34±3.66	0.006
LVEF (%)	62.83±3.45	64.10±1.89	0.135
LAD (mm)	35.91±5.58	38.10±5.71	0.175
Conventional doppler echocardiography			
E (cm/sec)	71.90±12.72	79.35±14.54	0.063
A (cm/sec)	75.21±14.99	68.19±13.03	0.074
E/A ratio	0.99±0.26	1.22±0.41	0.034
Deceleration time (msec)	194.72±38.82	164.20±32.17	0.003
IVRT (msec)	79.11±18.66	77.30±15.72	0.702
Diastolic dysfunction (n, %)	20, 55.6	5, 25	0.028
Tissue doppler echocardiography			
LV lateral annulus			
E_m_ (cm/sec)	11.22±3.09	13.68±2.84	0.005
A_m_ (cm/sec)	13.63±4.23	12.80±3.57	0.440
S_m_ (cm/sec)	8.38±1.89	9.60±1.84	0.024
E/E_m_	6.76±1.78	5.92±1.06	0.058
RV lateral annulus			
E_m_ (cm/sec)	13.62±4.57	14.47±3.79	0.462
A_m_ (cm/sec)	14.74±3.48	15.62±2.27	0.263
S_m_ (cm/sec)	11.98±3.08	11.88±3.54	0.914
2D speckle tracking			
Global longitudinal strain (GLS) (%)	−17.33±3.25	−20.35±2.61	0.001

Abbreviations: LVM, Left Ventricular Mass; LVMI, Left Ventricular Mass Index; IVSd, Interventricular septum thickness end diastole; LVPWd, Left ventricular posterior wall thickness end diastole; LVEDd, Left Ventricular End-Diastolic Diameter; LVEF, Left Ventricular Ejection Fraction; LAD, Left Atrium Diameter; E, Early Diastolic Mitral Inflow Velocities; A, Late Diastolic Mitral Inflow Velocities; IVRT, Isovolumetric Relaxation Time; E_m_, Early Myocardial Diastolic Velocity; A_m_, Late Myocardial Diastolic Velocity; S_m_, Systolic Myocardial Velocity.

In tissue Doppler measurements, the left ventricular early myocardial diastolic velocity (E_m_) and lateral systolic myocardial velocity (S_m_) in patients with PTC were significantly lower than the BNG group (11.22 ± 3.09 cm/sec, 13.68 ± 2.84 cm/sec, P = 0.005; 8.38 ± 1.89 cm/sec, 9.60 ± 1.84 cm/sec, P = 0.024, respectively). Other tissue Doppler variables were similar between the groups (Table 2).

The evaluation of the myocardial tissue properties with 2D-STE imaging demonstrated that the group of the PTC had significantly impaired GLS as compared with the group of the BNG (−17.33 ± 3.25, −20.35 ± 2.61, P = 0.001, respectively) (Table 2).

### Integrin αvβ3 and TNF-α

**Fig. 1:**
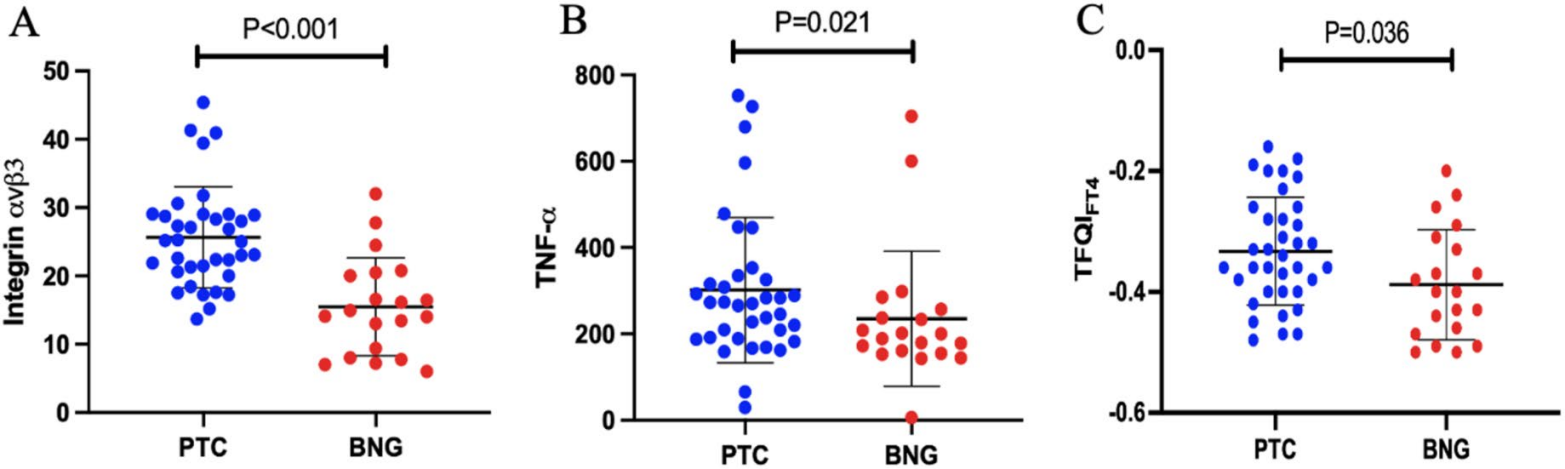
Comparison of integrin αvβ3, TNF-α, and TFQI_FT4_ between PTC patients and patients with BNG. Scatter diagrams A-C show the comparisons of integrin αvβ3, TNF-α, and TFQI_FT4_ between PTC patients and patients with BNG respectively. Thick black lines represent the mean value and thinner black lines represent the standard deviation for all parameters.

The integrin $\rm{\alpha }$ v $\upbeta$ 3 and TNF- $\rm{\alpha }$ concentrations were significantly higher in the PTC group than in BNG subjects (24.27 ± 8.01 ng/mL, 15.47 ± 7.18 ng/mL; 271.68 ng/L (29.5–752.5.5.5), 194.5 ng/L (6.11–704.07.11.07); respectively, P < 0.001) (Figure 1). In addition, the integrin $\rm{\alpha }$ v $\upbeta$ 3 and TNF- $\rm{\alpha }$ concentrations in the PTC patients with lymph node metastasis (n=14) were also statistically significantly higher than those in the PTC patients without lymph node metastasis (29.52 ± 7.85 ng/mL, 23.16 ± 6.08 ng/mL, P = 0.017; 313.84 ng/L (29.52–752.03.52.03), 228.95 ng/L (65.43–596.11.43.11), P = 0.019, respectively).

### Index of central thyroid hormone sensitivity

The PTC patients had significantly higher levels of TFQI_FT4_ compared to the BNG subjects (Figure 1).

### Correlations

There was a positive correlation between integrin $\rm{\alpha }$ v $\upbeta$ 3 concentration and TNF- $\rm{\alpha }$ (r = 0.652, P < 0.001), TFQI_FT4_ (r = 0.271, P = 0.043), and GLS (r = 0.306, P = 0.022), whereas age was negatively correlated with integrin $\rm{\alpha }$ v $\upbeta$ 3 concentration (r = −0.0396, P = 0.002) in the whole group. In addition, there was a positive correlation between GLS and TFQI_FT4_ (r = 0.275, P = 0.040). However, TNF-α levels were not significantly correlated with GLS or echocardiographic parameters of systolic or diastolic function. Furthermore, no significant correlations were observed between GLS or diastolic dysfunction and tumor-related characteristics such as tumor size, multifocality, minimal extrathyroidal extension, lymph node metastasis, and stage.

### Regression analyses

**Fig. 2:**
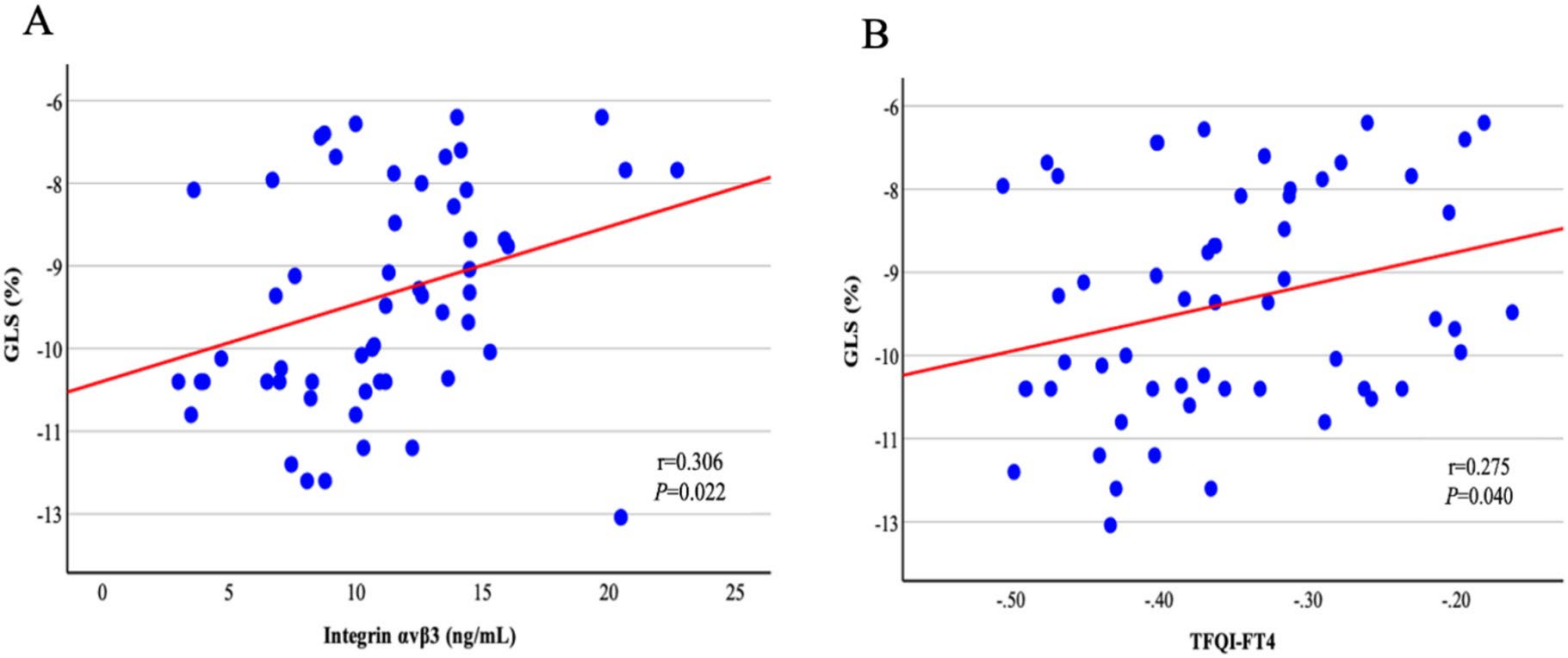
Integrin αvβ3 and TFQI_FT4_ are associated with impaired GLS. Integrin αvβ3 is plotted on the x-axis, with GLS on the y-axis (**A**) and TFQI_FT4_ is plotted on the the x-axis, with GLS on the y-axis (**B**).

Serum integrin αvβ3 levels were associated with GLS values in multivariable linear regression analyses performed in the entire study population (Table 3). Similarly, TFQI_FT4_ was also associated with GLS (Table 4). A scatter plot illustrating the relationship between integrin αvβ3 levels and TFQI_FT4_ with average GLS values is presented in Figure 2. Moreover, as shown in Table 5, integrin αvβ3 levels demonstrated a significant association with the presence of LV diastolic dysfunction in logistic regression analysis [P = 0.026, RR (95% CI) = 1.101 (1.012–1.194)].

**Table 3:** Multivariable linear regression analysis between GLS and Integrin ${\boldsymbol{\upalpha}}$ v ${\boldsymbol{\upbeta}}$ 3.

	Multivariable linear regression analysis						
	B	Standard error	Standardized coefficients beta	95% CI		t	*P* value
				Lower	Upper		
Age	0.071	0.041	0.259	−0.012	0.154	1.721	0.091
Gender	−0.312	1.071	−0.040	−2.462	1.838	−0.292	0.772
BMI	0.039	0.082	0.064	−0.126	0.204	0.471	0.640
Integrin $\rm{\alpha }$ v $\upbeta$ 3	0.153	0.054	0.399	0.043	0.262	2.801	**0.007**

R: 0.398, R2: 0.158, Adjusted R2: 0.092, F: 2.401, p: 0.062, Durbin-Watson: 2.366.

**Table 4:** Multivariable linear regression analysis between GLS and TFQI_FT4_.

	Multivariable linear regression analysis						
	B	Standard error	Standardized coefficients beta	95% CI		t	*P* value
				Lower	Upper		
Age	0.029	0.039	0.104	−0.049	0.107	0.737	0.464
Gender	−0.409	1.096	−0.052	−2.609	1.792	−0.373	0.711
BMI	0.099	0.083	0.162	−0.068	0.266	1.186	0.241
TFQI_FT4_	10.800	4.797	0.299	1.171	20.430	2.252	0.029

R: 0.342, R2: 0.117, Adjusted R2: 0.048, F: 1.686, p: 0.168, Durbin-Watson: 2.385.

**Table 5:** The associations between integrin ${\boldsymbol{\upalpha}}$ v ${\boldsymbol{\upbeta}}$ 3 level and LV diastolic dysfunction in the binary logistic regression analysis.

Variable	B	*P* value	RR	95 % CI
Age	0.054	0.085	1.056	0.993 to 1.123
Gender	−1.470	0.063	0.230	0.049 to 1.084
BMI	−0.022	0.695	0.978	0.876 to 1.092
Integrin $\rm{\alpha }$ v $\upbeta$ 3	0.097	0.026	1.101	1.012 to 1.199

## Discussion

In the present study, we identified subclinical myocardial dysfunctions in treatment-naive euthyroid patients with PTC in the absence of overt CVD. Using sensitive echocardiographic techniques, including tissue Doppler imaging and two-dimensional speckle-tracking echocardiography, PTC patients exhibited impaired diastolic function parameters and reduced GLS compared with individuals with BNG. These findings indicate that subtle myocardial functional differences may be detectable at the time of PTC diagnosis, prior to therapeutic interventions. In parallel, circulating integrin αvβ3 levels and TFQI_FT4_ levels were higher in PTC patients, and both markers were associated with impaired GLS.

While DTC typically carries a favorable prognosis, with increasing incidence and long-term survival expected, studies suggest that mortality in these patients is also attributed to causes other than thyroid cancer itself^5^. Previous studies have demonstrated that deaths from non-thyroid malignancies represent 30.1%–31.1% of mortality, thyroid cancer-specific deaths account for 22.8% to 46.4%, and CV-related deaths constitute 9.8%–21.3% of cases^28–30^. These findings highlight the importance of developing a more comprehensive understanding of the factors contributing to CVDs in patients with thyroid cancer. Most prior studies assessing CV risks in DTC patients have focused on those undergoing TSH suppression therapy, employing observational designs that often exclude treatment-naive patients^5,6,31,32^. However, despite these methodological limitations, the existing literature consistently supports the presence of both systolic and diastolic dysfunction in DTC patients. Taillard et al.^33^ reported that moderate TSH suppression therapy is associated with diastolic dysfunction, while Abdulrahman et al.^34^ documented both systolic and diastolic impairments in patients receiving long-term therapy. In this context, our findings suggest that early subclinical myocardial alterations may be present even in patients who have yet received any treatment, including TSH suppression therapy. Relatively small changes in GLS, even when values fall within borderline or conventionally normal ranges, should not be considered clinically trivial, as speckle-tracking-derived GLS has been consistently shown to detect early subendocardial myocardial dysfunction^35,36^. In this regard, the recent American Heart Association scientific statement emphasizes the superior ability of GLS to identify subclinical myocardial dysfunction and to provide incremental prognostic information beyond conventional echocardiographic measures^36^. Consistent with our findings, Abdelrazka et al.^37^ demonstrated that patients with subclinical thyroid dysfunction exhibit impaired GLS despite preserved EF, supporting the concept that GLS is a sensitive marker of early, subclinical myocardial dysfunction. In addition, no statistically significant associations were observed between tumor-related variables and either GLS or the presence of diastolic dysfunction in our study. The indolent nature of PTC, heterogeneity in disease duration, and the relatively limited sample size may have reduced to detect more subtle relationships between tumor characteristics and cardiac findings. Consequently, our findings should not be interpreted as establishing direct prognostic implications, but rather as identifying early functional differences that may be relevant in the context of integrin-mediated thyroid–cardiac interactions. Future prospective studies incorporating long-term cardiovascular outcomes will be essential to determine whether these subclinical echocardiographic changes translate into clinically meaningful risk in this specific patient population.

Our study demonstrated that circulating integrin αvβ3 levels were significantly higher in patients with PTC compared to those with benign thyroid disease and that higher circulating integrin αvβ3 levels were associated with impaired GLS and the presence of diastolic dysfunction.. Integrin αvβ3, a plasma membrane receptor abundantly expressed on malignant thyroid cells and proliferating vascular endothelium, has previously been implicated in tumor progression and CV remodeling^14,15,19^. Experimental studies have shown that integrin αvβ3 is expressed on cardiac myofibroblasts and macrophages involved extracellular matrix (ECM) turnover, as well as on vascular endothelial cells during angiogenesis, and that its activation promotes fibroblast proliferation, migration, and differentiation, ultimately contributing to myocardial fibrosis and collagen deposition^15,38^. Mechanistically, integrin αvβ3 functions as a mechanosensitive receptor linking ECM remodeling to inflammatory signaling via focal adhesion kinase-dependent pathways and downstream nuclear factor-KB activation, thereby facilitating the expression of pro-inflammatory cytokines^15^. In addition, integrin αvβ3 has been reported to initiate non-genomic signaling via pathways such as MAPK/ERK, PI3K/Ak, and focal adhesion kinase (FAK), which may modulate cardiomyocyte contractile function, cytoskeletal organization, and intracellular calcium homeostasis^15,18^. Consistent with this framework, we observed significantly elevated TNF-α levels in patients with PTC, with particularly pronounced increases in both TNF-α and integrin αvβ3 in cases with lymph node metastasis. Although TNF-α levels were higher in patients with PTC, no direct association was observed between TNF-α and cardiac parameters. Taken together, these findings indicate that future clinical-experimental studies are required to clarify the clinical significance of subclinical CV alterations in treatment-naive PTC patients.

To evaluate central thyroid hormone sensitivity, the TFQI was first described by Laclaustra and colleagues^20^ who demonstrated that impaired TFQI was significantly associated with type 2 diabetes and diabetes-related mortality. Subsequent studies have reported that impaired sensitivity of the HPT axis to THs may be linked to various adverse clinical conditions, including type 2 diabetes^20^, gestational diabetes^39^, non-alcoholic fatty liver disease (NAFLD)^40^, and CVDs^41^. Using data from the National Health and Nutrition Examination Survey (NHANES) 2007-2012, Hui et al.^22^ demonstrated that among individuals with normal thyroid function, TFQI levels were significantly higher in those with CVD, and that TFQI was independently and positively associated with CVD prevalence in multivariable analyses. In accordance with previous literature^21,42^, our study demonstrated significantly elevated TFQI_FT4_ levels in PTC patients compared to individuals with BNG. This finding suggests that central thyroid hormone sensitivity is compromised in PTC patients. Moreover, we demonstrated associations between central thyroid hormone resistance and both impaired GLS and elevated integrin αvβ3 levels. Given that TFQI_FT4_ should be interpreted as a state marker of central thyroid hormone sensitivity rather than a measure of thyroid hormone excess or deficiency, these findings suggest that alterations in central thyroid hormone sensitivity in patients with PTC may coexist with markers of subclinical myocardial dysfunction and circulating integrin αvβ3 levels. However, the cross-sectional design of the study precludes inference regarding the causal direction of the relationship between TFQI_FT4_ alterations and myocardial dysfunction.

This study has several limitations, yet these do not undermine its significance as a novel contribution to the literature. First, the relatively small sample size may limit the generalizability of our findings. However, despite the limited cohort, the study provides pioneering insights into the relationship between elevated integrin αvβ3 levels, impaired central thyroid hormone sensitivity, and subclinical cardiac dysfunction in treatment-naive PTC patients, which has not been reported previously. The study was conducted at a tertiary referral center with extensive surgical experience in thyroid cancer. All participants were consecutively recruited from patients scheduled for thyroid surgery. The size of the benign control group was determined by the availability of surgically treated benign cases meeting strict matching criteria during the study period. Second, the cross-sectional design prevents us from establishing causal relationships between these parameters and CV outcomes. Longitudinal studies are needed to elucidate the long-term impact of these findings on CV morbidity and mortality. Third, brain natriuretic peptide (BNP) or N-terminal proBNP were not measured in this study, precluding biochemical assessment of myocardial wall stress and heart failure phenotype. Although cardiometabolic profiles were comparable between groups, residual effects of treated or subclinical hypertension and dyslipidemia on GLS cannot be fully ruled out and represent a limitation of this observational study. An important limitation of the present study is the reliance on circulating integrin αvβ3 levels without direct correlation to tissue expression. Although integrin αvβ3 is known to be expressed in thyroid cells and cardiomyocytes, the absence of paired serum–tissue analyses from available surgical specimens in the papillary thyroid carcinoma group limits confirmation of tissue-specific expression. Moreover, the lack of direct myocardial tissue assessment precludes definitive conclusions regarding the precise role of integrin αvβ3 in the observed cardiac phenotype. In addition, the observational design of the study restricts causal inference, and the associations identified should be interpreted within this context. Despite these limitations, our findings provide clinically relevant evidence of a systemic association between circulating integrin αvβ3 and cardiac functional parameters, supporting its potential role as a biologically meaningful mediator at the thyroid–heart interface and underscoring the need for future studies integrating serum biomarkers with tissue-level and mechanistic investigations.

In conclusion, treatment-naive, euthyroid patients with PTC exhibit subclinical myocardial dysfunction characterized by impaired GLS and diastolic abnormalities, despite preserved EF and absence of overt CVD. Elevated circulating integrin αvβ3 levels and altered central thyroid hormone sensitivity were associated with impaired GLS, while integrin αvβ3 was also related to diastolic dysfunction. These findings suggest that integrin αvβ3 and central thyroid hormone sensitivity indices may help identify vulnerability to subclinical myocardial dysfunction in newly diagnosed PTC patients. Future large-scale, longitudinal studies integrating clinical outcomes are required to determine the prognostic relevance and biological basis of these associations.

## Data Availability

Some or all datasets generated during or analyzed during the current study are not publicly available but are available from the corresponding author on reasonable request.
